# Change in non-alcoholic beverage sales following a 10-pence levy on sugar-sweetened beverages within a national chain of restaurants in the UK: interrupted time series analysis of a natural experiment

**DOI:** 10.1136/jech-2017-209947

**Published:** 2017-10-16

**Authors:** Laura Cornelsen, Oliver T Mytton, Jean Adams, Antonio Gasparrini, Dalia Iskander, Cecile Knai, Mark Petticrew, Courtney Scott, Richard Smith, Claire Thompson, Martin White, Steven Cummins

**Affiliations:** 1 Faculty of Public Health & Policy, London School of Hygiene & Tropical Medicine, London, UK; 2 Centre for Diet & Activity Research & MRC Epidemiology Unit, University of Cambridge School of Clinical Medicine, Institute of Metabolic Science, Cambridge, UK

**Keywords:** sugar-sweetened beverages, diet, sugar tax, natural experiment, interrupted-time series

## Abstract

**Background:**

This study evaluates changes in sales of non-alcoholic beverages in Jamie’s Italian, a national chain of commercial restaurants in the UK, following the introduction of a £0.10 per-beverage levy on sugar-sweetened beverages (SSBs) and supporting activity including beverage menu redesign, new products and establishment of a children’s health fund from levy proceeds.

**Methods:**

We used an interrupted time series design to quantify changes in sales of non-alcoholic beverages 12 weeks and 6 months after implementation of the levy, using itemised electronic point of sale data. Main outcomes were number of SSBs and other non-alcoholic beverages sold per customer. Linear regression and multilevel random effects models, adjusting for seasonality and clustering, were used to investigate changes in SSB sales across all restaurants (n=37) and by tertiles of baseline restaurant SSB sales per customer.

**Results:**

Compared with the prelevy period, the number of SSBs sold per customer declined by 11.0% (−17.3% to −4.3%) at 12 weeks and 9.3% (−15.2% to −3.2%) at 6 months. For non-levied beverages, sales per customer of children’s fruit juice declined by 34.7% (−55.3% to −4.3%) at 12 weeks and 9.9% (−16.8% to −2.4%) at 6 months. At 6 months, sales per customer of fruit juice increased by 21.8% (14.0% to 30.2%) but sales of diet cola (−7.3%; −11.7% to −2.8%) and bottled waters (−6.5%; −11.0% to −1.7%) declined. Changes in sales were only observed in restaurants in the medium and high tertiles of baseline SSB sales per customer.

**Conclusions:**

Introduction of a £0.10 levy on SSBs alongside complementary activities is associated with declines in SSB sales per customer in the short and medium term, particularly in restaurants with higher baseline sales of SSBs.

## Introduction

Consumption of sugar-sweetened beverages (SSBs) is associated with obesity, type 2 diabetes, cardiovascular disease and dental caries.^1–5^ In the UK, an adult consumes an average of 50 kcal per day and children consume an average of 100 kcal from SSBs.^6^ SSBs may account for half of the excess calories consumed per day by children.^7^ Randomised controlled trials show that decreasing consumption of SSBs can reduce weight gain and body mass index in children and adolescents.^6 7^ Reducing intakes of SSBs has therefore been identified as an important in improving cardiometabolic health.^3 8^

In recent years, there has been increasing interest in the use of fiscal measures as instruments to reduce the consumption of SSBs as part of wider population-based strategies to prevent non-communicable disease.^9–11^ However, there is limited primary evidence on the effectiveness of fiscal measures on either the sale or consumption of SSBs.^9^ Research in this area mainly comprises modelling studies, laboratory experiments and small field studies in cafeterias in schools and workplaces.^7 9^ State-level regulation is relatively rare, with evaluations of these rarer still, but where excise taxes on SSBs have been introduced and evaluated, such as in Mexico or Berkeley, California, they have been associated with short-term reductions in SSB purchases.^10 12–18^

**Figure 1:**
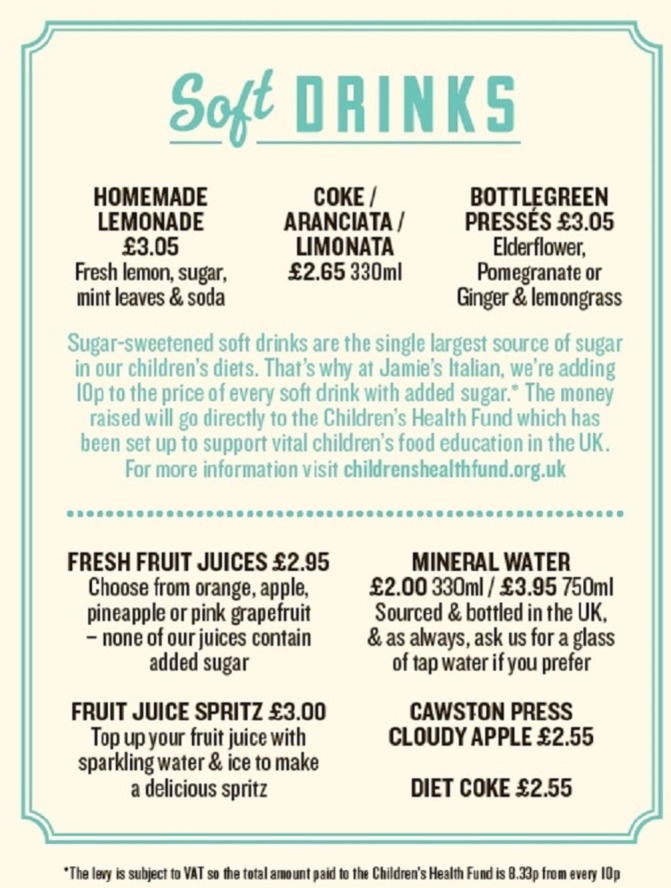
Non-alcoholic section of the beverage menu. Reproduced with permission by Jamie’s Italian Restaurants.

On the 1 September 2015, Jamie’s Italian, a national chain of UK restaurants, added a £0.10 levy to the price of non-alcoholic SSBs sold within them. The prelevy price of SSBs ranged from £2.60 to £3.25 (excluding the levy), thus addition of the levy equated to a price increase of 3.1%–3.8%. Other non-alcoholic beverages were similar in price, ranging from £2.00 and £3.95. In combination with the introduction of the levy, Jamie’s Italian reorganised the non-alcoholic beverage menu into two sections: SSBs and other beverages (juices, bottled waters and diet cola). In addition, fruit spritzers (fruit juice mixed with water) were added to the main non-alcoholic beverage menu. Text (see figure 1) on the SSB section of the non-alcoholic beverage menu explained the decision to implement the levy and that proceeds from the levy would go directly to a Children’s Health Fund that offered grants for children’s health initiatives. Introduction of the levy was supported by a Channel 4 television documentary ‘Jamie’s Sugar Rush’, first broadcast on 3 September 2015.^19^ Thus, the levy can be seen as a complex ‘intervention’ comprising a fiscal component (a price rise) in combination with other non-fiscal components that could plausibly help reduce purchases of SSBs.

We take advantage of this natural experiment to assess whether the implementation of the levy in combination with non-price activities (for convenience referred to as the ‘intervention’) is associated with changes in sales of non-alcoholic beverages. We use an interrupted time series (ITS) design to compare the number of SSBs, and other non-alcoholic beverages, sold per customer before implementation of the intervention with sales at 12 weeks and 6 months after. We use itemised electronic point of sale (EPOS) data and investigate the effect of the intervention across all restaurants and whether this effect varies by tertiles of baseline restaurant SSB sales per customer.

## Methods

In evaluations of natural experiments, the researcher has no control over the design and delivery of the intervention.^20^ The intervention was implemented in all restaurants simultaneously by Jamie’s Italian for logistical and commercial reasons and was outside the control of the research team. For this study, randomisation was therefore not possible and as no suitable control condition existed, a pragmatic evaluation using an ITS design^21^ was employed. ITS approaches are considered as the strongest design available in such contexts and have been widely used in evaluations of public health interventions.^12^ Here, we use Jandoc *et al*’s recommended guidance for the transparent reporting of studies using ITS designs.^22^

### Data

Itemised time-series data on the number of all non-alcoholic beverages sold in each of 37 eligible restaurants were extracted from the Jamie’s Italian EPOS system (MICROS Retail Systems) and provided to the authors by the company. To be eligible for inclusion, restaurants had to be operating between 23 June 2014 and 28 February 2016. To assess short-term (12 weeks) changes in sales, we used restaurant-aggregated itemised weekly data on the number of beverages sold from 8 June 2015 to 22 November 2015 in 37 restaurants. This equated to a time series of n=24 weeks, with 12 weeks of data either side of the implementation date of the intervention. To assess medium-term changes in sales at 6 months, we analysed the itemised number of beverages sold over 23 periods of 4 weeks each, starting from 23 June 2014 to 28 February 2016, from each of the 37 restaurants. These 4 weekly periods are standard company accounting periods, and we used data from the maximum number of periods available through the EPOS system. As the implementation date (1 September 2015) of the levy fell in the middle of a 4-week accounting period, data for that period were split into 2-week periods to allow for the specification of an exact implementation date in analyses. The numbers of beverages sold were standardised by adjusting for the number of customers visiting each restaurant. To do this, we divided the number of beverages sold by the number of ‘covers’ for each restaurant (one cover is equal to one adult or child customer) in each period, including the period split in two.

Non-alcoholic beverages were separated into two main groups: on-menu and off-menu. On-menu refers to beverages present on the restaurant beverage menu (see figure 1). Off-menu refers to beverages that could be requested by the customer but were not listed on the menu. For the on-menu group, beverages were split into five categories: (1) SSBs subject to the levy, (2) fruit juices, (3) fruit juices on the children’s menu, (4) diet cola and (5) bottled water (see online supplementary appendix 1). Beverages not listed on the menu were split into two categories: (6) SSBs subject to the levy and (7) ‘mixers’, which were not levied. In addition, separate categories were created for fruit spritz (a mix of water and fruit juices), beverages, introduced to the main menu when the intervention was implemented and children’s ‘fruity water’ and milk, introduced to the children’s menu in June 2015. This categorisation allowed assessment of the main effect of the intervention on sales of SSBs and exploration of possible substitution effects. Data on tap water orders were not recorded through the EPOS system. A further 1.3% of all drinks sold during the study period were excluded as they did not fit the categories above (eg, slim-line tonics, smoothies and cordial drinks).

10.1136/jech-2017-209947.supp1 Supplementary file 1

First, changes in the number of non-alcoholic beverages sold per customer across all restaurants at 12 weeks and 6 months were explored. Second, these analyses were stratified by tertiles of baseline restaurant on-menu SSB sales per customer (low: n=12 restaurants; medium: n=13 restaurants; and high: n=12 restaurants) and assessed at 6 months only. Online supplementary appendix 2 lists the locations of included restaurants.

### Statistical analysis

Changes in non-alcoholic beverage sales associated with the intervention in the ITS were modelled as an immediate step change in the log-transformed number of beverages sold per customer. The step change was defined as a categorical variable equal to zero before implementation (up to 31 August) and one afterwards. This assumes an immediate and stable effect of the intervention.^21^ We did not analyse changes in trends as the postintervention period was too short for meaningful interpretation.

Estimates using weekly data were adjusted for time trend in number of beverages sold per customer and estimates using 4 weekly data additionally adjusted for quarterly seasonal effects (see online supplementary appendix 3). Analyses of aggregated weekly data were conducted using simple linear regression as data were only available at the aggregate level (ie, for 37 restaurants combined). Analyses of 4 weekly data were conducted using a hierarchical multilevel model with restaurant-specific random effects in intercept and trend. We report robust standard errors to account for residual autocorrelation and clustering. Magnitude and statistical significance of the coefficient of the categorical variable was used to assess the changes associated with the intervention (see online supplementary appendix 3 for details on the model). Estimated coefficients were exponentiated and expressed as a percentage change in the number of beverages sold per customer. Sensitivity analyses were conducted that tested the robustness of the timing of the effect by varying the date of implementation to the month preceding and following introduction of the intervention (see online supplementary appendix 5).

## Results

In the 12 months prior to the introduction of the intervention 2 058 581 non-alcoholic beverages were sold within study-eligible restaurants, of which 38% (n=775 230) were SSBs. Table 1 shows the mean number of beverages sold per 4-weekly period in each category per restaurant and per customer. Before and after the introduction of the intervention, SSBs were the most commonly purchased non-alcoholic beverage per restaurant and per customer, followed by bottled water and diet cola. On a per-customer basis, after introduction of the intervention, an increase in the mean number of fruit juices (main menu) sold and reductions in the SSB, diet cola and children’s fruit juice categories were observed. Table 2 reports the mean number of newly introduced beverages (fruit spritz, children’s fruity water and children’s milk) sold per 4-weekly period by restaurant and by customer. Compared with existing beverages, overall sales of these new beverages were low.

**Table 1:** Mean number of beverages sold per restaurant and per customer (4-weekly sales periods)

	Mean number of beverages sold (SD) per restaurant		Mean number of beverages sold (SD) per customer	
Beverage category	Preintervention, per 4-weekly sales period (n=561 periods)	Postintervention, per 4-weekly sales period (n=259 periods)	Preintervention, per 4-weekly sales period (n=561 periods)	Postintervention, per 4-weekly sales period (n=259 periods)
On menu				
Levied SSBs	1781 (1269)	1425 (820)	0.174 (0.043)	0.155 (0.036)
Fruit juice (main menu)	408 (1053)	379 (821)	0.030 (0.030)	0.033 (0.029)
Fruit juice (children’s menu)	450 (349)	289 (182)	0.045 (0.024)	0.033 (0.018)
Diet cola	732 (445)	613 (337)	0.073 (0.020)	0.067 (0.020)
Bottled water	979 (1427)	828 (1192)	0.083 (0.044)	0.077 (0.047)
Off menu				
Levied SSBs	300 (192)	273 (171)	0.030 (0.013)	0.030 (0.015)
Mixers	300 (250)	302 (251)	0.029 (0.013)	0.031 (0.016)

Analyses are based on n=820 restaurant periods: prelevy n=561, postlevy n=259.

SSBs, sugar-sweetened beverages.

**Table 2:** Mean number of newly introduced non-alcoholic beverages sold following implementation

	Mean number of beverages sold postintervention per restaurant/period (SD) (n=259)	Mean number of beverages sold postintervention per customer/period (SD) (n=259)
Fruit spritzers (main menu)	122 (75)	0.014 (0.008)
Children’s fruity water	65 (44)	0.008 (0.005)
Children’s milk	41 (31)	0.005 (0.003)

Analyses are for 4-weekly sales periods. Fruit spritz was introduced to menu 1st of September. Children’s fruity water and milk were introduced to children’s menu in June 2015.

### Changes in sales of SSBs

Table 3 reports the percentage change in the mean number of beverages sold per customer at 12 weeks and 6 months after introduction of the intervention across all restaurants, compared with the preintervention period (see online supplementary appendix 4 for graphs of time series data). At 12 weeks, the intervention was associated with an 11.04% (95% CI −17.30% to −4.30%; p=0.003) decrease in the mean number of on-menu SSBs sold per customer compared with the 12-week period preceding the intervention, adjusting for trend. At 6 months, change in sales was smaller, with the intervention associated with a 9.34% (95% CI −15.21% to −3.15%; p=0.004) decrease in mean number of on-menu SSBs sold per customer. No changes in sales of off-menu SSBs were observed. Changes in sales at 6 months differed by tertile of baseline restaurant SSB sales per customer. Table 4 shows that the intervention was associated with a 12.8% (95% CI −19.27% to −5.82%; p=0.001) reduction in the number of on-menu SSBs sold per customer in high SSB sales restaurants and a reduction of 16.47% (95% CI −20.55% to −12.19%; p<0.001) in medium SSB sales restaurants, compared with the preintervention period. No changes in sales were observed in low SSB sales restaurants. In medium SSB sales restaurants, introduction of the intervention was associated with a 11.40% (95% CI −17.30% to −4.97%; p=0.001) fall in the number of off-menu SSBs sold per customer, but no association was found in either low or high SSB sales restaurants.

**Table 3:** Percentage change in the number of beverage sales per customer at 12 weeks and 6 months following implementation

Beverage category	Change at 12 weeks*			Change at 6 months†		
On-menu	Change (%)	95**%** CI	p Value	Change (%)	95**%** CI	p Value
Levied SSBs	−11.04	(−17.30 to 4.30)	p=0.003	−9.34	(−15.21 to 3.15)	p=0.004
Fruit juice (main menu)	3.36	(−4.50 to 11.96)	p=0.394	21.77	(14.00 to 30.21)	p<0.0001
Fruit juice (children’s menu)	−34.69	(−55.43 to 4.30)	p=0.031	−9.88	(−16.81 to 2.37)	p=0.011
Diet cola	−1.88	(−6.01 to 2.53)	p=0.377	−7.32	(−11.66 to 2.76)	p=0.002
Bottled water	5.44	(−1.19 to 12.64)	p=0.104	−6.48	(−11.04 to 1.69)	p=0.009
**Off-menu**						
Levied SSBs	−8.06	(−16.39 to 1.21)	p=0.084	−4.21	(−10.24 to 2.12)	p=0.187
Mixers	21.53	(8.22 to 36.62)	p=0.002	2.43	(−3.15 to 8.33)	p=0.402

Analyses at 12 weeks are based on n=888 restaurant weeks; analyses at 6 months based on n=820 restaurant periods.

Percentages are the exponentiated coefficients of change in log-transformed numbers of beverages sold per customer and are calculated from: $\%\Delta \hat{y}=100*\left[\exp\left({\hat{\beta }}_{2}\right)-1\right]$ (see online supplementary appendix 3 for more details).

*Robust standard errors.

†Robust clustered standard errors adjusted for seasonality and clustering.

SSBs, sugar-sweetened beverages.

**Table 4:** Percentage change in the number of beverages sold per customer at 6 months following implementation by tertiles of baseline restaurant SSB sales per customer

Beverage category	Restaurants with low SSB sales per customer*			Restaurants with medium SSB sales per customer*			Restaurants with high SSB sales per customer*		
On-menu	Change (%)	**95%** CI	p Value	Change (%)	**95%** CI	p Value	Change (%)	**95%** CI	p Value
Levied SSBs	3.05	(−12.54 to 21.41)	p=0.720	−16.47	(−20.55 to 12.19)	p<0.001	−12.80	(−19.27 to 5.82)	p=0.001
Fruit juice (main menu)	34.99	(18.77 to 53.42)	p<0.001	12.52	(4.92 to 20.68)	p<0.001	19.96	(5.65 to 36.21)	p=0.005
Fruit juice (children’s menu)	−7.13	(−19.35 to 6.93)	p=0.007	−7.96	(−22.28 to 8.98)	p=0.338	−14.36	(−22.04 to 6.01)	p=0.001
Diet cola	−1.00	(−10.42 to 9.53)	p=0.853	−14.19	(−20.07 to 7.78)	p<0.001	−5.45	(−10.68 to 0)	p=0.049
Bottled water	−6.20	(−16.47 to 5.44)	p=0.282	−8.24	(−13.50 to 2.76)	p=0.004	−4.78	(−12.45 to 3.46)	p=0.249
**Off-menu**									
Levied SSBs	4.19	(−9.79 to 20.44)	p=0.574	−11.40	(−17.30 to 4.97)	p=0.001	−4.21	(−13.50 to 6.08)	p=0.411
Mixers	2.53	(−8.42 to 14.80)	p=0.664	1.41	(−6.48 to 9.86)	p=0.739	3.36	(−6.67 to 14.57)	p=0.525

Analyses at 6 months based on n=820 restaurant periods.

*Robust clustered standard errors adjusted for seasonality and clustering. Percentages are the exponentiated coefficients of change in log-transformed numbers of beverages sold per customer and are calculated from: $\%\Delta \hat{y}=100*\left[\exp\left({\hat{\beta }}_{2}\right)-1\right]$ (see online supplementary appendix 3 for more details).

SSBs, sugar-sweetened beverages.

### Changes in sales of other non-alcoholic beverages

Table 3 shows that the intervention was associated with increases and decreases in the number of non-levied beverages sold per customer at 12 weeks and 6 months, compared with the preintervention period. At 12 weeks, the intervention was associated with a 21.53% (95% CI 8.22% to 36.62%; p=0.002) increase in the number of off-menu mixers sold per customer and a 34.69% (95% CI −55.43% to −4.30%; p=0.031) decrease in the number of children’s menu fruit juices sold per customer. At 6 months, the intervention was associated with an increase of 21.77% (95% CI 14.00% to 30.21%; p≤0.0001) in the number of fruit juices (main menu) sold per customer. However, the number of children’s menu fruit juices sold per customer decreased by 9.88% (95% CI −16.81% to −2.37%; p=0.011), diet cola decreased by 7.32% (95% CI −11.66% to −2.76%; p=0.002) and bottled water decreased by 6.48% (95% CI −11.04% to −1.69%; p=0.009). Stratified analysis (table 4) showed that the intervention was associated with an increase in the mean number of fruit juice (main menu) beverages sold per customer across all tertiles, with the largest increase of 34.99% (95% CI 18.77% to 53.42%; p<0.001) observed in restaurants with the lowest baseline SSB sales per customer. There were decreases in the numbers of diet colas and bottled waters sold per customer, but associations were primarily observed in restaurants with medium baseline SSB sales. An association between the intervention and a decrease in the mean number of children’s fruit juices sold was observed in restaurants with the highest baseline SSB only (−14.36; 95% CI −22.04% to −6.01%; p=0.001).

Sensitivity analyses of the robustness of the timing of the changes associated with the intervention confirmed the strongest effects at the actual time of the implementation (see online supplementary appendix 5 for supplementary data).

## Discussion

The introduction of a the £0.10 levy on SSBs combined with other supporting activities in Jamie’s Italian restaurants was associated with an 11.04% decrease in the mean number of on-menu SSBs sold per customer at 12 weeks and a 9.34% decrease at 6 months. Decreases at 6 months were only observed in restaurants with medium and high baseline SSB sales per customer, suggesting that the intervention may be more effective in restaurants with higher underlying sales of SSBs. Our analyses show a step decrease in number of SSBs sold per customer, and sensitivity analyses suggests this decrease occurred at time of implementation. This locates our observed changes at the point of the intervention’s introduction and strengthens the case for it being the likely explanation for our observed changes, rather than a general secular decline or as a result of other unobserved external factors.

We observed a general decrease in numbers of non-alcoholic beverages sold per customer, with the exception of fruit juice, postintervention. This is counter to evaluations in other settings, such as Mexico, where there have been increases in sales of diet beverages and bottled water as substitutes for SSBs.^12^ Here, the provision of new beverage options and orders of tap water may have acted as substitutes and may partially account for the decline in sales of levied SSBs and other soft drinks. However, this is unlikely to be the sole explanation for the observed decline. Declines in SSBs sales were greater than declines in sales of other beverages, suggesting that other elements of the intervention (beyond provision of new beverage options) may be important.

### Comparison with literature on fiscal interventions targeting SSB consumption

The changes observed in this study are relatively large given the size of the levy. However, recent evidence from Berkeley, California,^18^ showed that changes of the magnitude reported here may be plausible even when price rises are relatively small (21% decrease in sales in response to a penny-per-ounce tax^18^). Economic modelling and other evaluations suggest that sales reductions are similar in magnitude to the price change (a price elasticity of 0.8–1.2).^12 23–27^ In this study, we observed a decline in sales of 10%–12% in response to an average price increase of 3.5%. While this difference could be due to price elasticities cited above being estimated using supermarket purchase data in more aggregated groupings and where such drinks are considerably cheaper, it could also suggest that changes observed here may not be entirely due to the price increase. A range of complementary activities were implemented alongside the levy, including beverage menu redesign, donation of proceeds to a children’s health fund, new beverages and widespread media coverage. The Jamie Oliver ‘brand’ is also associated with campaigning on food and health issues. These factors may have acted in combination with the price increase suggesting that multifaceted interventions with a fiscal measure at their core may plausibly affect behaviour and have greater effects than interventions comprised solely of a fiscal component.

### Importance of non-fiscal components for behaviour change

Research on the introduction of nominal taxes on single-use plastic bags suggests that psychological processes are important contributors to intervention effectiveness by modifying habitual behaviours through contextual change.^28–30^ In the case of plastic bags, contextual change comprises a nominal tax and a prompt at point of sale asking customers if they wish to purchase them. In economic studies of taxes on alcohol, price tags marked as ‘tax-included’ on the shelf reduces sales at a greater rate than when supplementary taxes are applied at the cash register only.^31^ Similar processes may be in operation here. In addition to the levy on SSBs (the price component), the non-price components can be seen as important elements of ‘contextual change’ that may prompt customers to adapt their behaviour either to avoid the levy for economic reasons or to bring their behaviour in line with underlying latent values.

The mean number of SSBs sold per customer was relatively small (0.174 beverages per customer) signifying that average absolute effects across the chain at 12 weeks and 6 months are correspondingly small. However, as the consumption of SSBs is patterned by individual-level factors, such as age and socioeconomic position,^26 32^ effects may be relatively larger in certain customer groups (such as children). As reductions observed here are per visit, repeat customers would likely see larger cumulative declines. As eating out of the home has increased over the last 40 years,^33^ the application of such interventions across the commercial restaurant sector could have meaningful population health impact.^34^

### Strengths and limitations

Our study has several strengths. First, we use itemised objective data on all non-alcoholic beverage sales to assess changes in numbers of drinks sold after the intervention was introduced. This allowed categorisation of individual beverages into appropriate analytical groups. Second, using a panel format, we were able to control for variations in customers in different restaurants via restaurant specific random effects over time. Third, we were able to analyse changes using weekly and 4-weekly data. Both showed robust associations between the intervention and decreases in SSB sales, accounting for underlying trend.

Limitations included being only able to adjust numbers of beverages sold by the overall number of customers, as disaggregated data on the number of adults and children visiting each restaurant are not routinely collected. Variations in the number of children visiting restaurants, beyond time and seasonal influences, may have biased estimates of changes in sales. Second, we could not model impacts using a price variable, as beverage prices did not vary across restaurants. Third, while for most restaurants the model fitted the 4-weekly data well, for three restaurants the fit was poorer. These were retained in the sample, as there was no indication that systematic errors were present in the data. As data are in 4-weekly periods that did not correspond to calendar months, we could not use external control variables to adjust for wider trends in beverage or hospitality sector sales. Finally, this study was undertaken in one chain of restaurants, limiting generalisability.

## Conclusion

There is consistent evidence that reducing consumption of SSBs would be beneficial to health.^1 2 14 15^ Fiscal measures could discourage SSB purchases, and even small price changes may plausibly reduce sales.^18 35^ Our findings suggest that the introduction of a small levy on SSBs combined with non-fiscal complementary activities is associated with declines in SSB sales when delivered at scale in a commercial restaurant setting. Further research evaluating such interventions in other restaurant settings, with a longer follow-up, is required to assess whether this is transferable, whether decreases in sales are sustained and whether reductions in sales translate into reductions in SSB consumption and to explore whether changes differ by population subgroups.

**What is already known on this subject**

- The WHO recently advocated the use of fiscal measures, such as taxes, to reduce the consumption of sugar-sweetened beverages (SSBs).
- While the potential impacts of SSB taxes on sales and consumption of SSBs have been estimated using modelling studies, primary studies evaluating the effects of such interventions delivered in a real-world settings are rare and have only been undertaken in countries with high consumption of SSBs (USA and Mexico).
- No evaluations of fiscal interventions have been undertaken in the UK where SSB consumption is lower or have been delivered at scale in commercial restaurant settings.

**What this study adds**

- At 12 weeks, sales per customer of SSBs had declined by 11% and at 6 months by 9.3% compared with the preintervention period. Reductions were the greatest in restaurants with higher SSBs sales per customer.
- The effects observed in this study were relatively large, which suggests that complementary activities associated with its introduction (eg, menu redesign, introduction of new beverages and information) may have the potential to augment changes induced by price increases.
