# Supplementary material for: Change in non-alcoholic beverage sales following a 10-pence levy on sugar-sweetened beverages within a national chain of restaurants in the UK: interrupted time series analysis of a natural experiment
Source: J Epidemiol Community Health. 2017 Oct 16;71(11):1107–12. doi: 10.1136/jech-2017-209947 (PMC5847096; doi:10.1136/jech-2017-209947)
Supplement: Supplementary file 1 [file jech-2017-209947supp001.pdf]

Supplementary material

Contents

1. **List of drinks** 2
2. **Regions** 3
3. **Models** 4
4. **Additional tables of results** 5
5. **Robustness to timing of the levy** 18

1. List of drinks

Table S1. List of drinks sold at Jamie's Italian restaurant during the study period

Levied SSBs	Fruit juice (main menu)	Fruit juice (children's menu)	Diet cola	Bottled water	Levied SSBs not on the menu	Mixer	Fruit juice and water mix	Children's fruity water	Children's milk	Other soft drinks (not on the menu)
Home Lemonade	Orange juice	Apple juice	Diet Coke	Still water	Cranberry juice	Tonic	Cawston apple	Fruity water	Organic milk	Light/slim tonic
Limonata	Apple juice	Orange juice		Sparkling water	Mango juice	Slim Tonic	Orange and spark.w		Refill milk	Milk
Aranciata	Pineapple juice	Refill (apple)		Soda water	Tonic	Tonic water	Apple and spark.w			Pomegranate juice
Bottlegreen Pressés	Grapefruit juice	Refill (orange)			Lemonade	Lemonade	Pineapple and spark.w			Slimline tonic
Coca Cola					Orange juice & lemonade	Bitter lemon	Grapefruit. and spark.w			Tomato juice
					Bitter lemon	Soda	Pomegranate. and spark.w			Strawberry smoothie
					Ginger beer	Coca Cola	Orange and still w.			Mango smoothie
					Ginger ale	Diet Coke	Apple and still w.			Red berry smoothie
					Lime & soda	Apple Juice	Pineapple and still w.			Forest fruit smoothie
					Blackcurrant & soda	Cranberry juice	Grapefruit and still w.			Orange & basil smoothie
					Lime cordial	Orange juice	Pomegranate and still w.			Feel Good juices
					Blackcurrant cordial	Pineapple juice				Orange juice and soda
					Orange cordial	Tomato Juice				Grapefruit juice and soda
					Ginger cordial	Pomegranate juice				
					Apple cordial	Grapefruit juice				
					Elderflower cordial	Mango juice				
					Dandelion cordial	Lime Cordial				
					Ice Tea	Ginger beer				
					Sprite	Dry Ginger				
					Chinotto	Ginger ale				
					Crodino					
					Pompelmo					
					Abbondio drinks					
					Claceau drinks					

2. Regions

Table S2. Regional classification of Jamie's Italian restaurants

London	Angel Islington, Bluewater, Canary Wharf, Covent Garden, Greenwich, London Bridge (opened in Apr 2015), Norwich, Piccadilly, Westfield, Westfield Stratford, Victoria (opened in May 2015)
South	Bath, Brighton, Bristol, Cambridge, Cardiff, Cheltenham, Exeter (opened in Jan 2015), Gatwick, Guildford, Kingston, Milton Keynes, Oxford, Portsmouth, Reading, St Albans
North	Aberdeen, Birmingham, Edinburgh, Glasgow, Harrogate, Leeds, Liverpool, Manchester, Newcastle, Nottingham, York

3. Models

a) Model to analyse the impact of levy on weekly number of beverages sold per customer

$$\ln(y_{jt}) = b_0 + b_1 time_t + b_2 levy_t + e_t$$

Where:

$\ln(y_{jt})$ is the log-transformed series of volume of number of beverages sold per customer in each of the drink categories (j=7) aggregated over 37 restaurants

$levy_t = 1\ after\ 1st\ of\ September\ and\ 0\ before$

$time_t\ is\ a\ linear\ time\ trend\ (t = 1, \ldots, 24\ weeks)$; 1 indicates week commencing 8th of June 2015

First order autocorrelation in the estimated model residuals was tested using Cumby-Huizinga test.

b) Model to analyse the impact of levy on four-weekly number of beverages sold per customer

$$\ln(y_{jit}) = b_0 + b_1 time_{it} + b_2 levy_{it} + b_3 S1_{it} + b_4 S2_{it} + b_5 S3_{it} + u_{0i} + u_{1i} time_{it} + e_{it}$$

Where in addition to the above,

$\ln(y_{jit})$ is the log-transformed series of volume of number of beverages sold per customer in each of the drink categories (j=7) in individual restaurants (i=37)

$S1 = 1\ if\ summer\ (23.06.14 - 14.09.14;\ 22.06.15 - 13.09.15); 0\ otherwise$
$S2 = 1\ if\ autumn\ (15.09.14 - 07.12.14; 14.09.15 - 06.12.15); 0\ otherwise$
$S3 = 1\ for\ winter\ (08.12.14 - 01.03.15; 07.12.15 - 28.02.16); 0\ otherwise$
(spring period: 02.03.15 – 21.06.15)

$u_{0i} - random\ intercept\ at\ restaurant\ level$

$u_{1i} - random\ slope\ at\ restaurant\ level$

$time_t\ is\ a\ linear\ time\ trend\ (t = 1, \ldots, 23\ four-weekly\ periods)$; 1 indicates week commencing c 23rd of June 2014

Data were tested for serial correlation using Wooldridge test for first-order autocorrelation.

4. Additional tables of results

Table S3. Weekly data (n=24 weeks)

	**Levied SSBs on menu**	**Fruit juice (main menu)**	**Fruit juice (children's menu)**	**Diet cola**	**Water**	**Levied SSBs off menu**	**Mixers**
Time	-0.002	-0.008	0.017	<0.0001	-0.012	0.004	-0.007
95% CI	[-0.009,0.005]	[-0.014,-0.001]	[-0.015,0.049]	[-0.003,0.004]	[-0.017,-0.007]	[-0.003,0.011]	[-0.017,0.002]
P-value	0.594	0.022	0.283	0.927	<0.0001	0.231	0.108
Levy_1Sept	-0.117	0.033	-0.426	-0.019	0.053	-0.084	0.195
	[-0.190,-0.044]	[-0.046,0.113]	[-0.808,-0.044]	[-0.062,0.025]	[-0.012,0.119]	[-0.179,0.012]	[0.079,0.312]
	0.003	0.394	0.031	0.377	0.104	0.084	0.002
Constant	-1.922	-3.261	-3.603	-2.897	-2.340	-3.729	-3.722
	[-1.986,-1.858]	[-3.312,-3.211]	[-3.848,-3.358]	[-2.931,-2.862]	[-2.377,-2.303]	[-3.782,-3.676]	[-3.794,-3.649]
	<0.0001	<0.0001	<0.0001	<0.0001	<0.0001	<0.0001	<0.0001
R^2	0.701	0.365	0.195	0.067	0.766	0.194	0.453
BIC	-70.5	-65.1	12.0	-88.2	-84.0	-70.0	-54.0
Autocorrelation at lag1[$]							
P-value	0.380	0.066	0.041	0.452	0.681	0.498	0.914

Notes: is the log-transformed series of volume of number of beverages sold per customer in each of the drink categories (j=7) aggregated over 37 restaurants; robust standard errors; [$]Cumby-Huizinga test for autocorrelation in first lag (test applied to models with non-robust SE's).

Table S4. Monthly data (all Jamie's Italian restaurants)

n=820 restaurant weeks	Levied SSBs on menu	Fruit juice (main menu)	Fruit juice (children's menu)	Diet cola	Water	Levied SSBs off menu	Mixers
Time	0.001	-0.006	-0.014	-0.002	<0.0001	0.001	-0.001
95% CI	[-0.003,0.005]	[-0.010,-0.002]	[-0.023,-0.005]	[-0.005,0.001]	[-0.005,0.005]	[-0.003,0.006]	[-0.006,0.003]
P-value	0.595	0.003	0.002	0.111	0.957	0.559	0.545
Levy_1Sept	-0.098	0.197	-0.104	-0.076	-0.067	-0.043	0.024
	[-0.165,-0.032]	[0.131,0.264]	[-0.184,-0.024]	[-0.124,-0.028]	[-0.117,-0.017]	[-0.108,0.021]	[-0.032,0.080]
	0.004	<0.0001	0.011	0.002	0.009	0.187	0.402
Sum.	0.050	0.009	-0.109	-0.042	0.023	0.053	-0.077
	[0.030,0.070]	[-0.025,0.044]	[-0.188,-0.029]	[-0.066,-0.018]	[-0.006,0.052]	[0.023,0.082]	[-0.112,-0.041]
	<0.0001	0.596	0.007	0.001	0.120	<0.001	<0.001
Aut.	-0.005	-0.015	-0.108	-0.006	0.004	0.002	0.011
	[-0.014,0.005]	[-0.030,-<0.0001]	[-0.135,-0.082]	[-0.016,0.004]	[-0.009,0.018]	[-0.011,0.015]	[-0.005,0.027]
	0.330	0.045	<0.0001	0.237	0.537	0.749	0.185
Wint.	-0.003	0.006	-0.008	0.011	-0.031	0.025	0.038
	[-0.008,0.003]	[-0.003,0.014]	[-0.025,0.009]	[0.003,0.018]	[-0.041,-0.022]	[0.014,0.035]	[0.025,0.051]
	0.302	0.210	0.349	0.007	<0.0001	<0.0001	<0.0001
Constant	-1.814	-3.624	-3.042	-2.635	-2.563	-3.631	-3.656
	[-1.893,-1.734]	[-3.759,-3.488]	[-3.217,-2.867]	[-2.723,-2.547]	[-2.689,-2.437]	[-3.761,-3.502]	[-3.789,-3.522]
	<0.001	<0.001	<0.001	<0.001	<0.001	<0.001	<0.001
BIC	-1084.783	-713.9	625.9	-1222.0	-834.9	-588.531	-225.9
p-value$	0.048	0.009	<0.001	0.595	0.002	0.066	0.004

Notes: Dependent variable is the log-transformed series of volume of number of beverages sold per customer in each of the drink categories; robust standard errors; $ Wooldridge test for autocorrelation in lag 1.

Table S5. Monthly data (Jamie's Italian restaurants in London)

London; n=230 restaurant weeks	Levied SSBs on menu	Fruit juice (main menu)	Fruit juice (children's menu)	Diet cola	Water	Levied SSBs off menu	Mixers
Time	<0.0001	-0.003	-0.016	-0.003	-0.001	0.005	-0.002
95% CI	[-0.006,0.005]	[-0.011,0.006]	[-0.044,0.011]	[-0.008,0.003]	[-0.008,0.006]	[-0.006,0.016]	[-0.012,0.009]
P-value	0.904	0.539	0.241	0.359	0.834	0.382	0.771
Levy_1Sept	-0.142	0.095	-0.093	-0.123	-0.092	-0.122	0.014
	[-0.219,-0.066]	[0.013,0.176]	[-0.303,0.117]	[-0.197,-0.049]	[-0.152,-0.032]	[-0.231,-0.013]	[-0.080,0.107]
	<0.0001	0.023	0.386	0.001	0.003	0.029	0.772
Sum.	0.044	-0.002	-0.029	-0.026	-0.035	0.062	-0.075
	[0.001,0.086]	[-0.082,0.079]	[-0.245,0.188]	[-0.056,0.005]	[-0.100,0.031]	[-0.005,0.130]	[-0.153,0.003]
	0.045	0.969	0.796	0.099	0.298	0.068	0.061
Aut.	-0.003	-0.007	-0.087	0.003	0.017	0.002	0.022
	[-0.023,0.018]	[-0.041,0.026]	[-0.156,-0.017]	[-0.019,0.025]	[-0.002,0.035]	[-0.030,0.034]	[-0.011,0.055]
	0.807	0.669	0.015	0.781	0.078	0.895	0.182
Wint.	-0.005	0.001	0.018	0.009	-0.025	0.022	0.042
	[-0.014,0.005]	[-0.020,0.022]	[-0.027,0.062]	[-0.005,0.024]	[-0.039,-0.011]	[-<0.0001,0.044]	[0.019,0.065]
	0.317	0.915	0.432	0.217	0.001	0.052	<0.0001
Constant	-1.762	-3.507	-3.137	-2.640	-2.210	-3.894	-3.765
	[-1.915,-1.610]	[-3.671,-3.343]	[-3.509,-2.765]	[-2.828,-2.451]	[-2.440,-1.980]	[-4.099,-3.689]	[-4.022,-3.508]
	<0.0001	<0.0001	<0.0001	<0.0001	<0.0001	<0.0001	<0.0001
bic	-314.4	-196.4	278.9	-348.3	-240.0	-73.2	-31.7
p-value$	0.814	0.012	0.026	0.365	0.003	0.12	0.008

Notes: Dependent variable is the log-transformed series of volume of number of beverages sold per customer in each of the drink categories; robust standard errors; $ Wooldridge test for autocorrelation in lag 1.

Table S6. Monthly data (Jamie's Italian restaurants in North)

North; n=253 restaurant weeks	Levied SSBs on the menu	Fruit juice (main menu)	Fruit juice (children's menu)	Diet cola	Water	Levied SSBs off the menu	Mixers (sold with alcohol)
Time	-0.002	-0.010	-0.020	-0.004	0.005	-0.002	0.005
95% CI	[-0.013,0.008]	[-0.017,-0.003]	[-0.035,-0.005]	[-0.009,0.001]	[-0.003,0.013]	[-0.012,0.007]	[-0.005,0.014]
P-value	0.668	0.007	0.008	0.094	0.233	0.664	0.315
Levy_1Sept	<0.0001	0.284	-0.097	-0.021	-0.122	0.017	-0.026
	[-0.187,0.186]	[0.158,0.411]	[-0.246,0.051]	[-0.130,0.088]	[-0.223,-0.020]	[-0.130,0.164]	[-0.144,0.092]
	0.997	<0.0001	0.200	0.708	0.019	0.824	0.665
Sum.	0.028	0.017	-0.210	-0.091	0.040	0.019	-0.073
	[-0.005,0.062]	[-0.040,0.074]	[-0.353,-0.068]	[-0.143,-0.038]	[0.005,0.075]	[-0.030,0.068]	[-0.124,-0.023]
	0.098	0.556	0.004	0.001	0.025	0.443	0.004
Aut.	-0.007	-0.028	-0.129	-0.018	0.004	0.008	0.011
	[-0.022,0.008]	[-0.056,0.001]	[-0.177,-0.081]	[-0.036,0.001]	[-0.021,0.029]	[-0.013,0.028]	[-0.020,0.042]
	0.372	0.056	<0.0001	0.065	0.732	0.462	0.491
Wint.	0.001	-0.001	-0.022	0.011	-0.039	0.032	0.040
	[-0.007,0.008]	[-0.012,0.011]	[-0.048,0.005]	[-0.003,0.025]	[-0.063,-0.015]	[0.014,0.049]	[0.020,0.060]
	0.869	0.926	0.115	0.126	0.001	<0.0001	<0.0001
Constant	-1.939	-3.826	-3.016	-2.573	-2.798	-3.263	-3.333
	[-2.065,-1.814]	[-3.974,-3.678]	[-3.419,-2.613]	[-2.698,-2.449]	[-2.900,-2.697]	[-3.493,-3.034]	[-3.498,-3.168]
	<0.0001	<0.0001	<0.0001	<0.0001	<0.0001	<0.0001	<0.0001
bic	-237.6	-200.8	260.2	-318.2	-228.7	-189.3	-113.0
p-value	0.256	0.550	0.001	0.473	0.368	0.039	0.434

Notes: Dependent variable is the log-transformed series of volume of number of beverages sold per customer in each of the drink categories; robust standard errors;
$ Wooldridge test for autocorrelation in lag 1

Table S7. Monthly data (Jamie's Italian restaurants in South)

South; n=337 restaurant weeks	Levied SSBs on menu	Fruit juice (main menu)	Fruit juice (children's menu)	Diet cola	Water	Levied SSBs off menu	Mixers
Time	0.004	-0.006	-0.009	<0.0001	-0.004	0.002	-0.006
95% CI	[0.001,0.008]	[-0.011,-<0.0001]	[-0.014,-0.004]	[-0.005,0.004]	[-0.012,0.004]	[-0.003,0.006]	[-0.010,-0.001]
P-value	0.023	0.033	0.001	0.817	0.311	0.532	0.023
Levy_1Sept	-0.138	0.207	-0.114	-0.081	-0.01	-0.03	0.066
	[-0.195,-0.082]	[0.096,0.318]	[-0.204,-0.024]	[-0.147,-0.014]	[-0.089,0.069]	[-0.109,0.049]	[-0.015,0.146]
	<0.0001	<0.0001	0.013	0.017	0.805	0.457	0.110
Sum.	0.069	0.009	-0.087	-0.019	0.050	0.069	-0.080
	[0.040,0.097]	[-0.042,0.059]	[-0.146,-0.027]	[-0.050,0.011]	[0.009,0.091]	[0.030,0.109]	[-0.141,-0.019]
	<0.0001	0.736	0.004	0.215	0.018	0.001	0.010
Aut.	-0.005	-0.012	-0.108	-0.005	-0.004	-0.003	0.003
	[-0.019,0.009]	[-0.030,0.007]	[-0.134,-0.083]	[-0.018,0.008]	[-0.028,0.021]	[-0.021,0.015]	[-0.021,0.027]
	0.501	0.225	<0.0001	0.482	0.763	0.762	0.791
Wint.	-0.004	0.013	-0.016	0.011	-0.029	0.021	0.034
	[-0.014,0.006]	[0.001,0.025]	[-0.038,0.006]	[-0.002,0.023]	[-0.041,-0.018]	[0.006,0.035]	[0.009,0.058]
	0.407	0.028	0.148	0.099	<0.0001	0.005	0.007
Constant	-1.754	-3.553	-2.997	-2.675	-2.635	-3.710	-3.819
	[-1.872,-1.637]	[-3.834,-3.273]	[-3.182,-2.811]	[-2.819,-2.530]	[-2.814,-2.456]	[-3.826,-3.594]	[-4.009,-3.629]
	<0.0001	<0.0001	<0.0001	<0.0001	<0.0001	<0.0001	<0.0001
BIC	-540.4	-280.4	113.6	-512.3	-345.9	-299.3	-31.2
p-value	0.015	0.007	0.128	0.501	0.011	0.813	0.037

Notes: Dependent variable is the log-transformed series of volume of number of beverages sold per customer in each of the drink categories; robust standard errors; [$] Wooldridge test for autocorrelation in lag 1.

Figure S1. Fitted vs actual values – weekly data

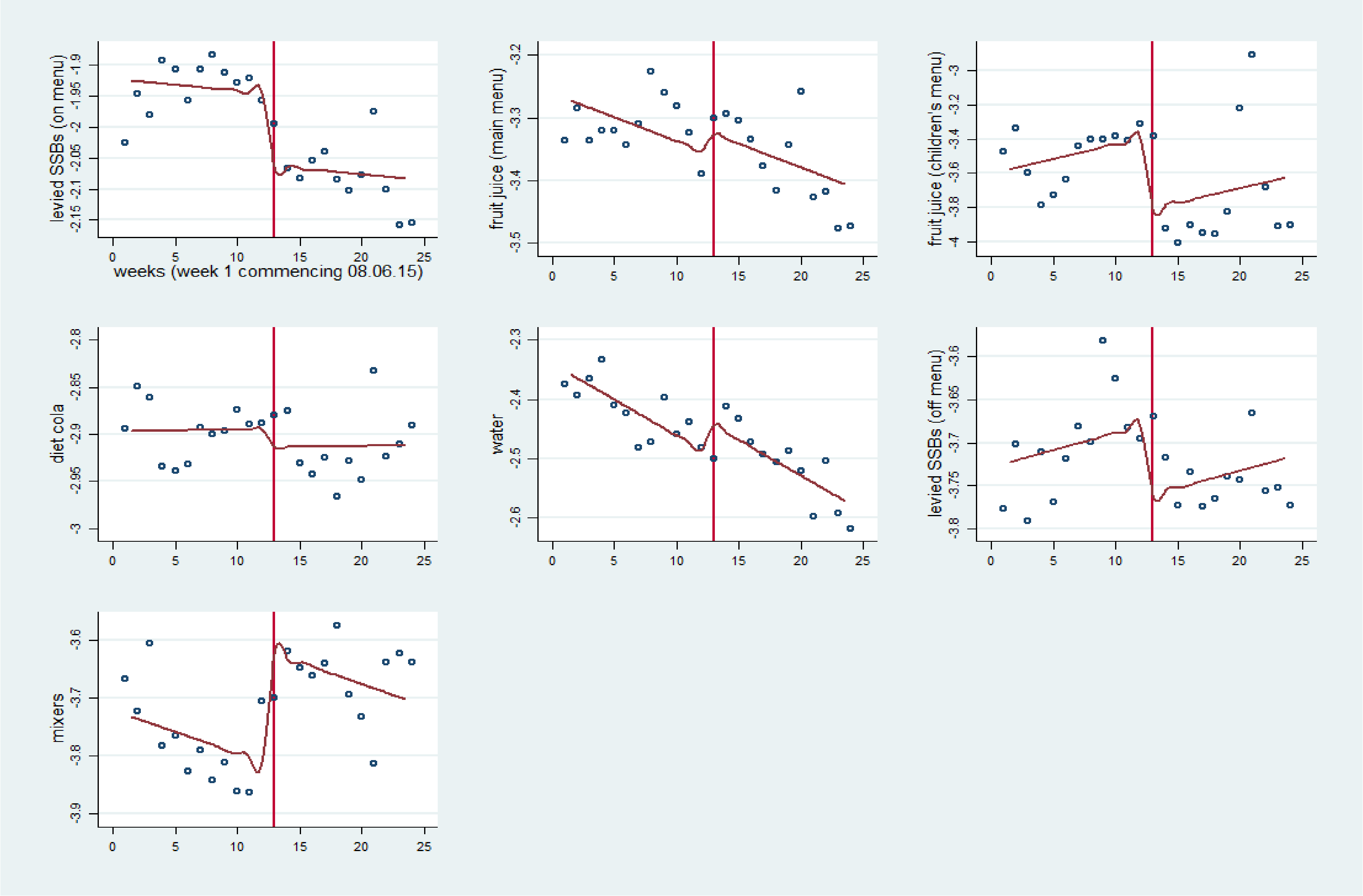

Figure S2. Fitted vs actual values - levied SSBs (on the menu); four-weekly data by restaurant

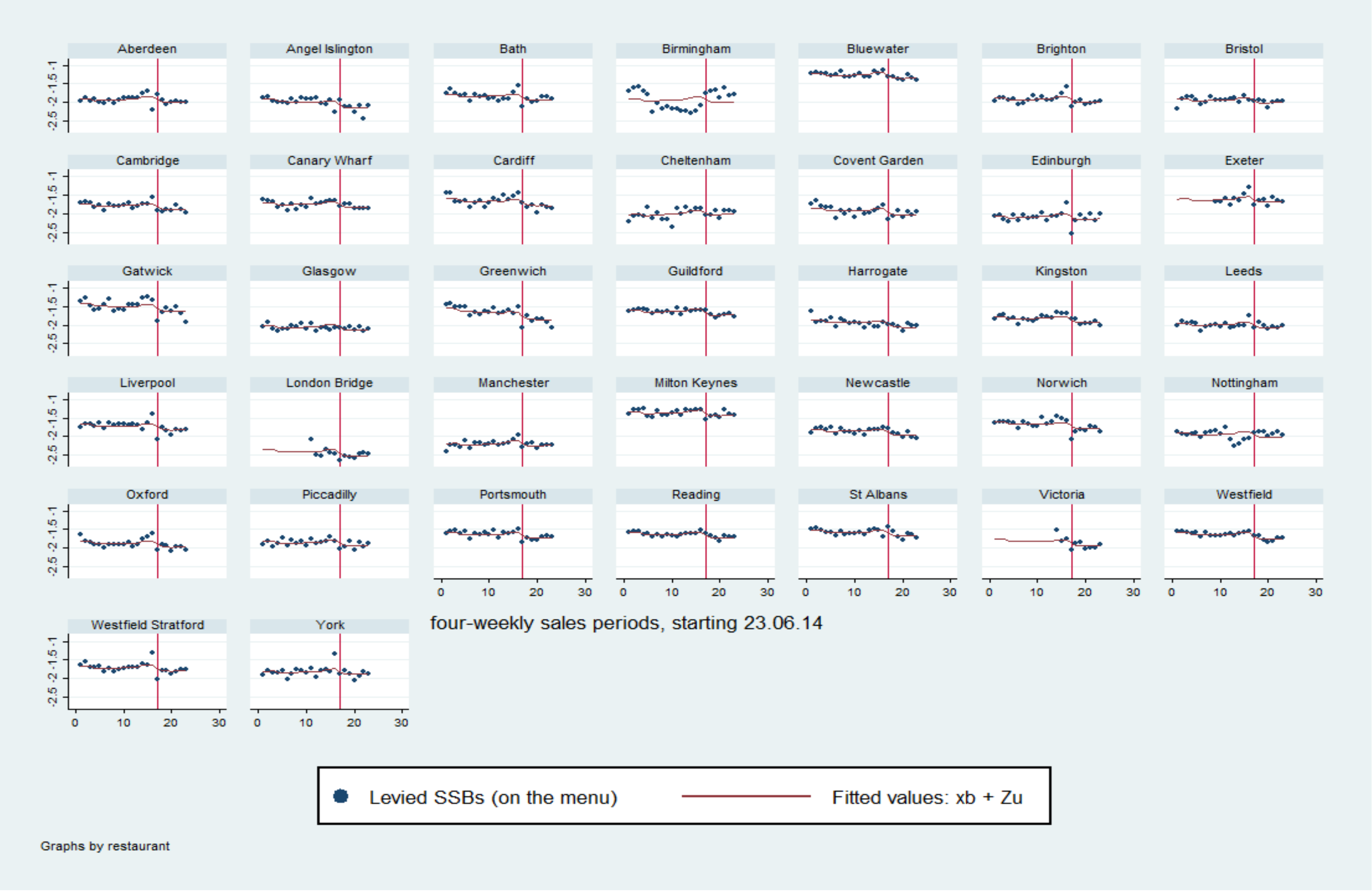

Figure S3. Fitted vs actual values – fruit juice (main menu); four-weekly data by restaurant

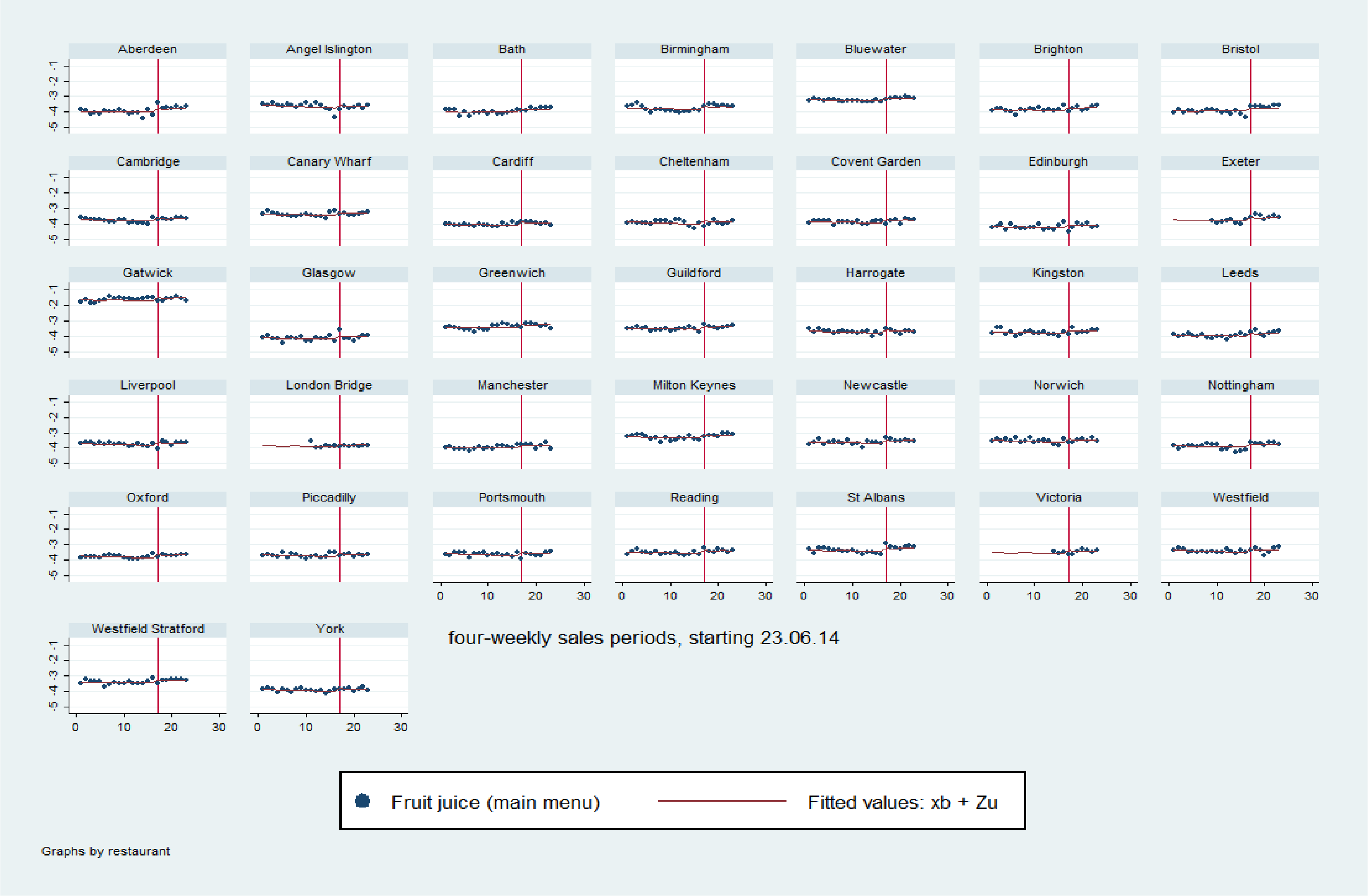

Figure S4. Fitted vs actual values – fruit juice (children's menu); four-weekly data by restaurant

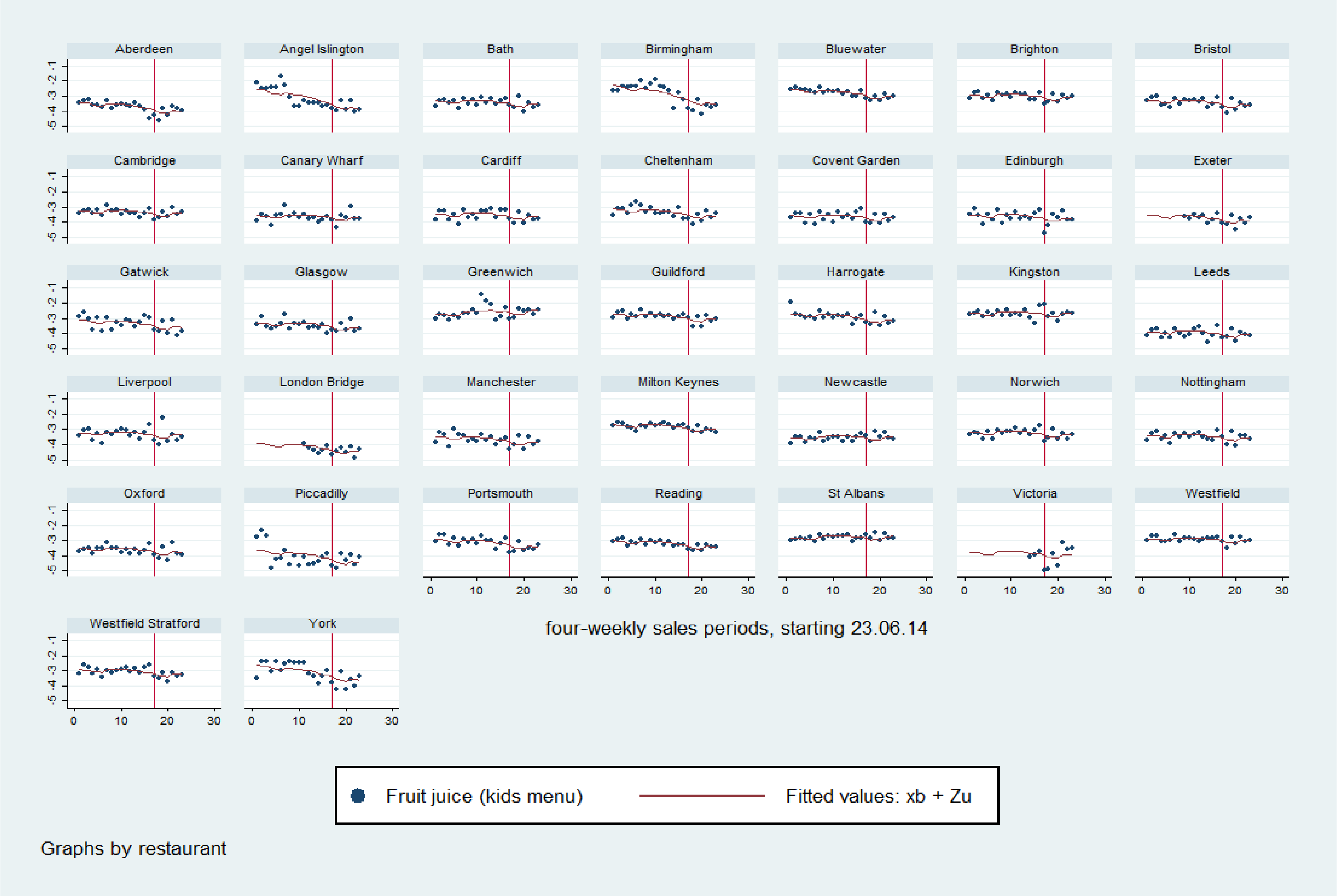

Figure S5. Fitted vs actual values – diet cola; four-weekly data by restaurant

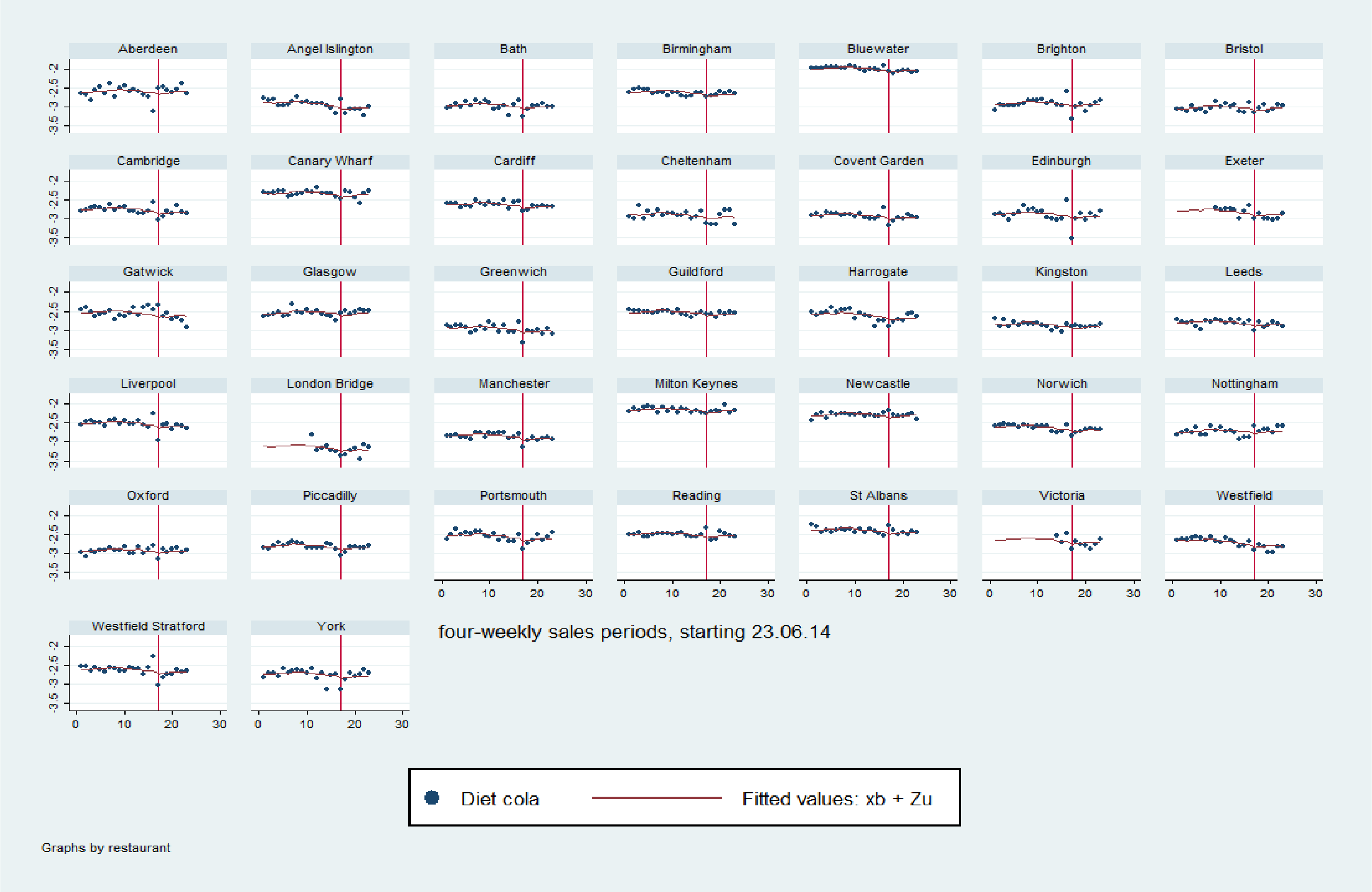

Figure S6. Fitted vs actual values – water; four-weekly data by restaurant

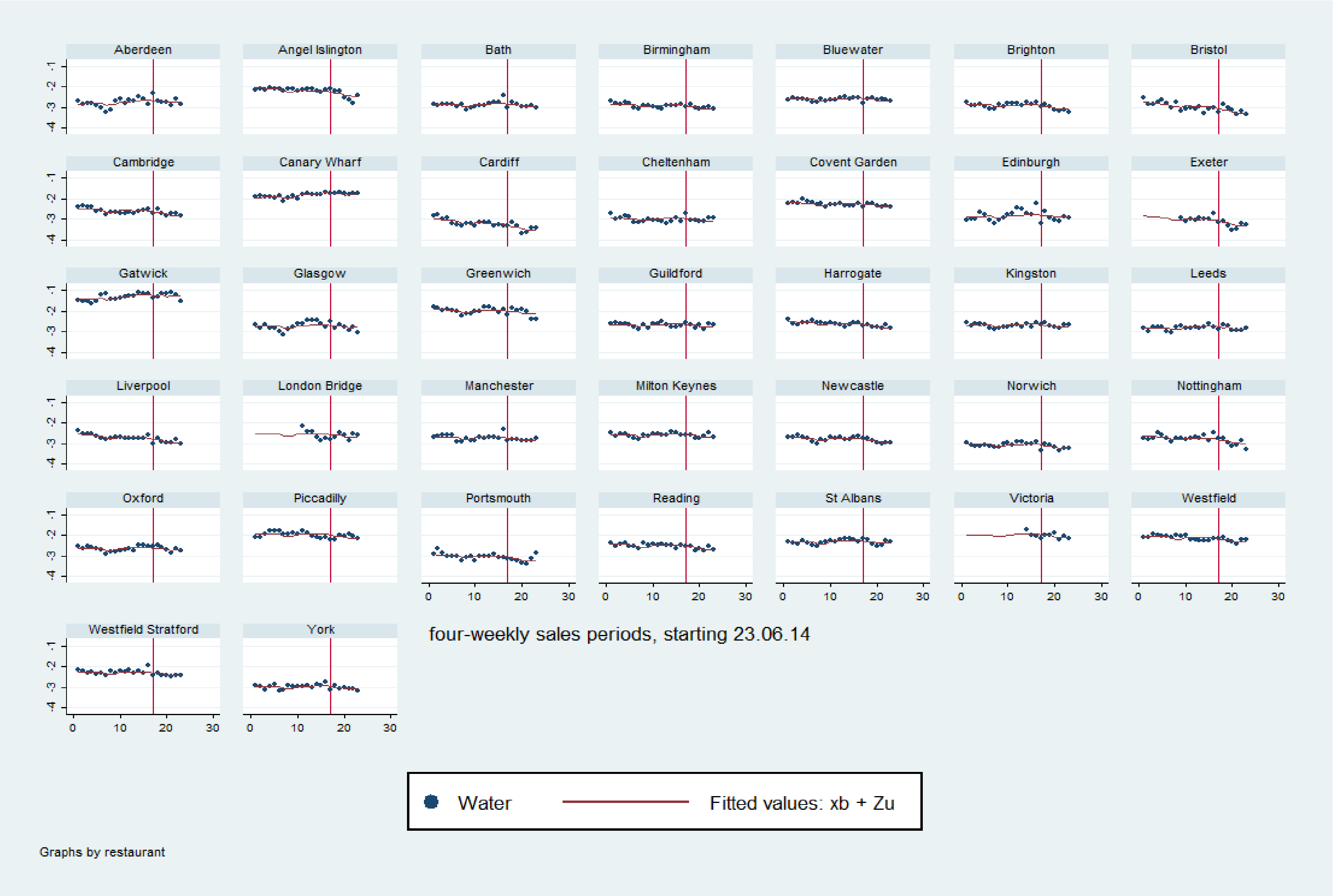

Figure S7. **Fitted vs actual values – levied SSBs (off menu); four-weekly data by restaurant**

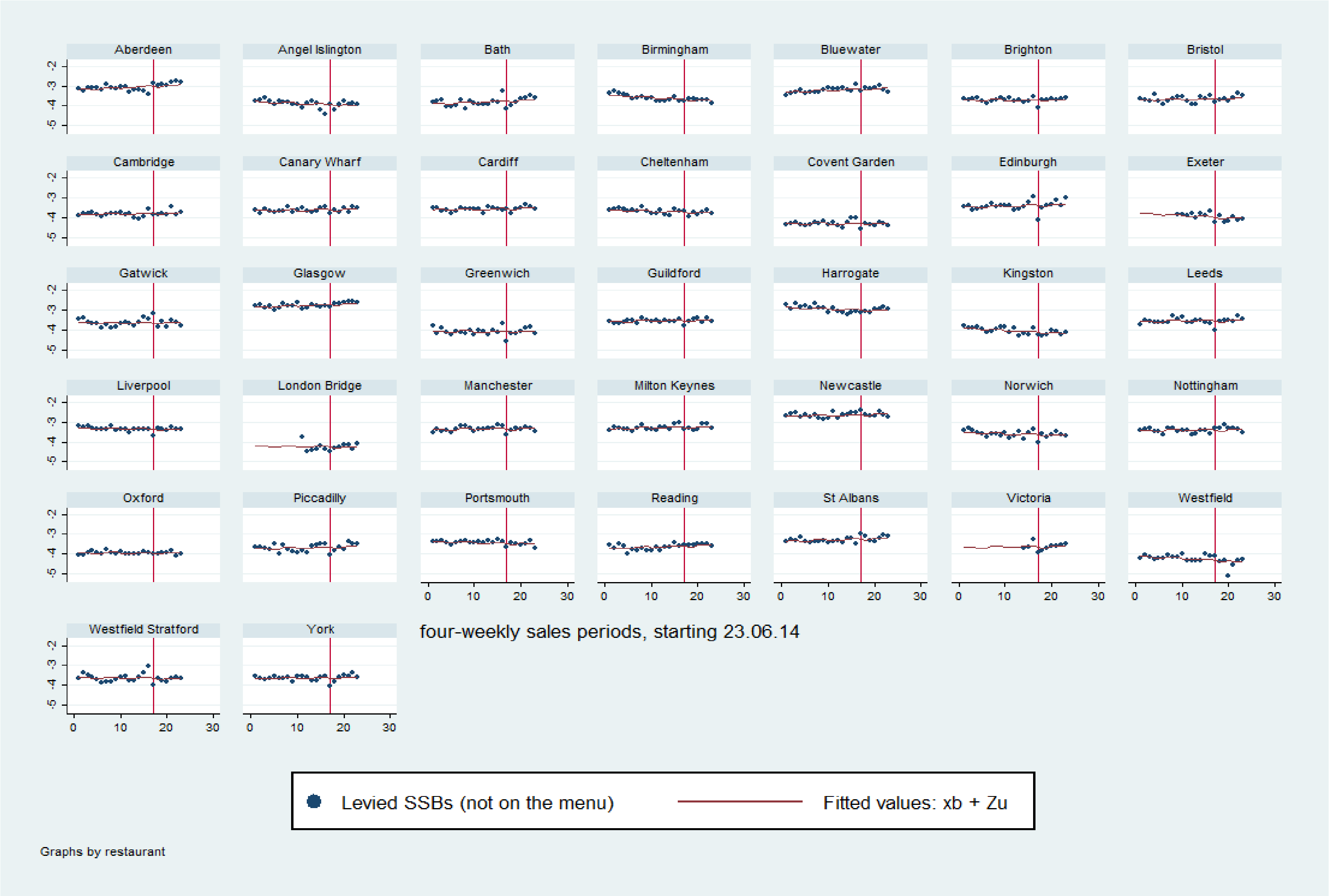

Figure S8. **Fitted vs actual values – mixers; four-weekly data by restaurant**

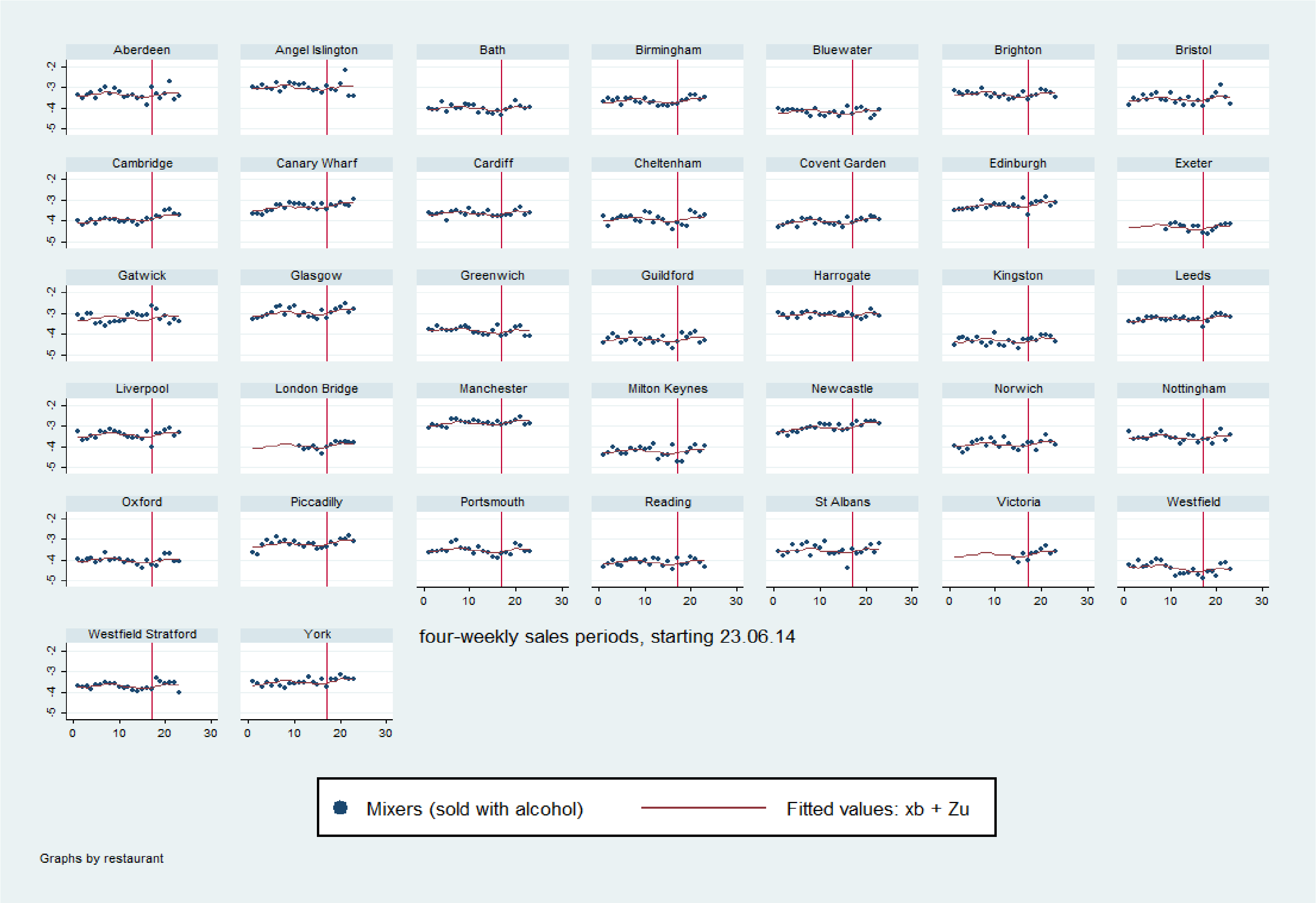

5. Robustness to timing of the levy

Robustness checks were undertaken with regard to the timing of intervention. First, we tested whether any effects on the volume of sales of levied SSBs (on-menu) were detected prior to the levy's introduction (from 17th of August), due to media coverage preceding the implementation of the levy. Secondly, we tested whether the effect from the levy could have been affected from coinciding with the beginning of the school year and thus more children visiting the restaurants. As we did not have exact figures on the number of children visiting the restaurants, more children visiting during certain period could have artificially increased the number of beverages sold by cover. This affects more weekly data as when modelling the four-weekly data we are able to control for seasonality as well as time effects. We test this by allowing the levy variable to equal to 1 (i.e. levy to take effect) from either 7th or 14th of September. Results showed that when using weekly data (table S8), the only model with statistically significant coefficient of the levy variable was one where the impact was allowed to start from the second week of September with a very similar effect size of -12.4% (Beta -0.124; 95% CI -18.7 to -6.1; P=0.001) reduction in sales. In 4-weekly data, the impact of the levy on the sales of SSBs (on the menu) reduced to -6.4% and -6.8% when the levy was assumed to take effect from mid-August or mid-September (Beta -0.064; 95% CI, -11.8 to -1; P=0.017, and Beta -0.068; 95% CI -11.6 to -2; P=0.005, respectively).

Table S8. Weekly data, robustness check for timing of the impact

	Levied SSBs on menu	Fruit juice (main menu)	Fruit juice (children's menu)	Diet cola	Water	Levied SSBs off menu	Mixers
Time	-0.007	-0.006	-0.003	-0.001	-0.012	0.001	-0.002
95% CI	[-0.015,0.001]	[-0.013,0.001]	[-0.037,0.031]	[-0.004,0.002]	[-0.016,-0.007]	[-0.006,0.008]	[-0.014,0.010]
P-value	0.101	0.102	0.850	0.512	<<0.00011	0.720	0.761
Levy_17Aug	-0.039	0.003	-0.107	0.001	0.046	-0.04	0.107
	[-0.140,0.061]	[-0.085,0.091]	[-0.534,0.320]	[-0.038,0.040]	[-0.019,0.111]	[-0.146,0.065]	[-0.058,0.271]
	0.425	0.945	0.608	0.967	0.154	0.436	0.191
Constant	-1.896	-3.27	-3.502	-2.891	-2.347	-3.712	-3.758
	[-1.964,-1.829]	[-3.317,-3.224]	[-3.719,-3.285]	[-2.921,-2.861]	[-2.379,-2.314]	[-3.763,-3.662]	[-3.846,-3.669]
	<<0.00011	<<0.00011	<<0.00011	<<0.00011	<<0.00011	<<0.00011	<<0.00011
Time	-0.004	-0.005	0.005	-0.001	-0.011	0.003	-0.008
	[-0.012,0.004]	[-0.012,0.002]	[-0.031,0.040]	[-0.004,0.003]	[-0.016,-0.006]	[-0.004,0.010]	[-0.017,0.001]
	0.343	0.128	0.784	0.760	<0.0001	0.399	0.086
Levy_24Aug	-0.087	-0.002	-0.231	-0.008	0.038	-0.066	0.203
	[-0.179,0.006]	[-0.090,0.086]	[-0.681,0.219]	[-0.049,0.033]	[-0.031,0.108]	[-0.167,0.034]	[0.100,0.305]
	0.064	0.962	0.298	0.686	0.264	0.186	0.001
Constant	-1.91	-3.271	-3.537	-2.893	-2.346	-3.721	-3.729
	[-1.977,-1.842]	[-3.322,-3.221]	[-3.779,-3.295]	[-2.925,-2.861]	[-2.381,-2.310]	[-3.773,-3.668]	[-3.805,-3.654]
	<0.0001	<0.0001	<0.0001	<0.0001	<0.0001	<0.0001	<0.0001
Time	-0.001	-0.006	0.026	0.001	-0.013	0.006	-0.007
	[-0.008,0.005]	[-0.012,0.001]	[0.001,0.050]	[-0.002,0.005]	[-0.017,-0.009]	[0.001,0.011]	[-0.016,0.003]
	0.644	0.075	0.039	0.506	<0.0001	0.026	0.151
Levy_7Sep	-0.124	0.006	-0.57	-0.035	0.073	-0.117	0.182
	[-0.187,-0.061]	[-0.079,0.090]	[-0.775,-0.365]	[-0.077,0.007]	[0.026,0.120]	[-0.186,-0.048]	[0.060,0.303]
	0.001	0.893	0	0.097	0.004	0.002	0.005
Constant	-1.929	-3.269	-3.664	-2.903	-2.332	-3.742	-3.719
	[-1.989,-1.869]	[-3.321,-3.218]	[-3.886,-3.443]	[-2.939,-2.866]	[-2.367,-2.296]	[-3.789,-3.696]	[-3.790,-3.648]
	<0.0001	<0.0001	<0.0001	<0.0001	<0.0001	<0.0001	<0.0001
Time	-0.004	-0.004	0.012	0.002	-0.01	0.006	-0.002
	[-0.011,0.004]	[-0.009,0.002]	[-0.019,0.043]	[-0.001,0.005]	[-0.016,-0.005]	[0.001,0.011]	[-0.013,0.008]
	0.306	0.202	0.439	0.163	0	0.022	0.643
Levy_14Sep	-0.089	-0.03	-0.353	-0.053	0.028	-0.116	0.117
	[-0.179,0.001]	[-0.110,0.050]	[-0.797,0.092]	[-0.083,-0.023]	[-0.043,0.099]	[-0.181,-0.051]	[-0.032,0.266]
	0.052	0.449	0.114	0.001	0.423	0.001	0.118
Constant	-1.92	-3.281	-3.604	-2.91	-2.345	-3.745	-3.737
	[-1.986,-1.854]	[-3.329,-3.233]	[-3.825,-3.383]	[-2.947,-2.872]	[-2.383,-2.307]	[-3.789,-3.701]	[-3.818,-3.656]
	<0.0001	<0.0001	<0.0001	<0.0001	<0.0001	<0.0001	<0.0001

Notes: Dependent variable is the log-transformed series of sales per cover for each of the drink categories; robust standard errors.

Table S9. Four-weekly data, robustness check for timing of the impact (impact starts from 17th of August)

n=783 restaurant weeks	Levied SSBs on menu	Fruit juice (main menu)	Fruit juice (children's menu)	Diet cola	Water	Levied SSBs off menu	Mixer
Time	-0.001	-0.007	-0.019	-0.007	-0.004	-0.002	-0.001
95% CI	[-0.005,0.002]	[-0.010,-0.003]	[-0.028,-0.010]	[-0.009,-0.004]	[-0.009,0.001]	[-0.006,0.002]	[-0.005,0.003]
P-value	0.501	<0.0001	<0.0001	<0.0001	0.079	0.386	0.522
Levy_17aug	-0.064	0.189	-0.060	0.011	-0.006	0.017	0.052
	[-0.118,-0.010]	[0.140,0.238]	[-0.142,0.023]	[-0.015,0.037]	[-0.043,0.030]	[-0.020,0.054]	[0.003,0.101]
	0.020	<0.0001	0.158	0.393	0.736	0.364	0.037
Sum.	0.071	0.016	-0.070	-0.057	0.009	0.056	-0.060
	[0.052,0.090]	[-0.021,0.052]	[-0.155,0.015]	[-0.081,-0.034]	[-0.024,0.041]	[0.024,0.088]	[-0.097,-0.024]
	<0.0001	0.406	0.104	<0.0001	0.597	0.001	0.001
Aut.	-0.001	-0.006	-0.026	-0.019	-0.028	0.009	0.038
	[-0.010,0.007]	[-0.022,0.009]	[-0.063,0.012]	[-0.030,-0.008]	[-0.042,-0.014]	[-0.002,0.020]	[0.021,0.055]
	0.768	0.441	0.177	<0.0001	<0.0001	0.097	<0.0001
Wint.	0.006	0.017	0.030	0.005	-0.034	0.026	0.029
	[0.001,0.010]	[0.009,0.026]	[0.009,0.050]	[-0.001,0.011]	[-0.045,-0.024]	[0.018,0.033]	[0.014,0.044]
	0.015	<0.0001	0.005	0.135	<0.0001	<0.0001	<0.0001
Constant	-1.812	-3.634	-3.084	-2.589	-2.510	-3.615	-3.670
	[-1.888,-1.736]	[-3.766,-3.501]	[-3.266,-2.902]	[-2.668,-2.510]	[-2.636,-2.385]	[-3.737,-3.494]	[-3.794,-3.547]
	<0.0001	<0.0001	<0.0001	<0.0001	<0.0001	<0.0001	<0.0001
BIC	-1214.4	-812.2	595.5	-1472.8	-889.9	-788.2	-293.1

Notes: Dependent variable is the log-transformed series of sales per cover for each of the drink categories; robust standard errors.

Table 7. Four-weekly data, robustness check for timing of the impact (impact starts from 14th of Sept.)

n=783	Levied SSBs on menu	Fruit juice (main menu)	Fruit juice (children's menu)	Diet cola	Water	Levied SSBs off menu	Mixers
Time	-0.002	-0.005	-0.014	-0.006	-0.004	-0.002	-0.001
95% CI	[-0.005,0.002]	[-0.008,-0.002]	[-0.023,-0.006]	[-0.008,-0.004]	[-0.009,0.001]	[-0.006,0.002]	[-0.005,0.002]
P-value	0.335	0.001	0.001	<0.0001	0.100	0.338	0.472
Levy_14Sept	-0.068	0.188	-0.158	-0.003	-0.018	0.023	0.062
	[-0.116,-0.020]	[0.142,0.234]	[-0.234,-0.083]	[-0.031,0.025]	[-0.058,0.022]	[-0.020,0.067]	[0.016,0.109]
	0.005	<0.0001	<0.0001	0.851	0.378	0.291	0.009
Sum.	0.062	0.044	-0.060	-0.053	0.010	0.057	-0.055
	[0.043,0.080]	[0.009,0.079]	[-0.142,0.021]	[-0.075,-0.031]	[-0.021,0.042]	[0.026,0.088]	[-0.090,-0.019]
	<0.0001	0.013	0.146	<0.0001	0.532	<0.0001	0.002
Aut.	<0.0001	-0.007	-0.006	-0.016	-0.026	0.008	0.035
	[-0.008,0.007]	[-0.023,0.009]	[-0.040,0.028]	[-0.027,-0.005]	[-0.040,-0.011]	[-0.003,0.019]	[0.020,0.051]
	0.952	0.399	0.746	0.004	0.001	0.155	<0.0001
Wint.	0.007	0.015	0.038	0.006	-0.033	0.025	0.028
	[0.002,0.011]	[0.007,0.024]	[0.019,0.058]	[-0.001,0.012]	[-0.044,-0.023]	[0.017,0.033]	[0.013,0.043]
	0.004	0.001	<0.0001	0.075	<0.0001	<0.0001	<0.0001
Constant	-1.809	-3.649	-3.135	-2.598	-2.516	-3.613	-3.669
	[-1.884,-1.734]	[-3.786,-3.513]	[-3.310,-2.960]	[-2.677,-2.519]	[-2.642,-2.390]	[-3.736,-3.490]	[-3.791,-3.546]
	<0.0001	<0.0001	<0.0001	<0.0001	<0.0001	<0.0001	<0.0001
BIC	-1218.8	-820.5	584.7	-1472.0	-890.9	-789.1	-295.4

Notes: Dependent variable is the log-transformed series of sales per cover for each of the drink categories; robust standard errors.
